# Antibacterial interactions between two monofloral honeys and several topical antiseptics, including essential oils

**DOI:** 10.1186/s12906-022-03695-x

**Published:** 2022-08-26

**Authors:** Brayden H. Gray, Kathryn J. Green, Robbie R. Haines, Katherine A. Hammer

**Affiliations:** 1 School of Biomedical Sciences, The Marshall Centre for Infectious Diseases Research and Training, The University of Western Australia, Crawley, WA Australia; 2 Cooperative Research Centre for Honey Bee Products Limited (CRC HBP), The University of Western Australia, Crawley, WA Australia

**Keywords:** *Apis mellifera*, Monoterpenes, Volatile oils, Topical therapy, Apitherapy, Antagonism, Minimum inhibitory concentration

## Abstract

**Background:**

Honey has broad spectrum antibacterial activity against clinically important organisms and may be suitable for treating superficial bacterial infections. However, very little data are available describing potential interactions between honey and other topically applied agents such as antiseptics or essential oils.

**Methods:**

Interactions between pairs of antibacterial agents were investigated by performing checkerboard assays and determining the fractional inhibitory concentration indices (FICIs). Interactions between the two monofloral honeys marri (from *Corymbia calophylla*) and manuka, and the antiseptic agents benzalkonium chloride, chlorhexidine digluconate, silver (I) nitrate, tea tree oil, and *Eucalyptus polybractea* oil were investigated against *Staphylococcus aureus* ATCC® 43300 and *Pseudomonas aeruginosa* ATCC® 27853.

**Results:**

Additive or indifferent interactions (FICI 0.5—2) were observed for all combinations against both organisms tested, with the exception of chlorhexidine and honey. Chlorhexidine and marri honey showed an antagonistic relationship against *S. aureus* (median FICI 2.00, range 1.25—4.83). Similarly, chlorhexidine and manuka honey showed antagonism against *S. aureus* (median FICI 2.33, range 2.00—2.67).

**Conclusions:**

With the exception of chlorhexidine, these data indicate that honey does not interfere with the antimicrobial activity of the tested agents, and that honey may be suitable for combination therapy with other topically applied antibacterial agents for treating superficial bacterial infections.

**Supplementary Information:**

The online version contains supplementary material available at 10.1186/s12906-022-03695-x.

## Background

With a global increase in infections caused by antibiotic resistant bacteria, there is renewed interest in alternative agents, such as honey, as potential therapeutic agents [1]. In fact, honey has been put into clinical practice in some parts of the world for limited indications [2]. The antibacterial mechanism of honey is both multifactorial and complex, with data suggesting that it is due to a combination of factors, including osmotic shock, low pH, protein denaturation, and oxidising agents [3]. Furthermore, each of these factors may have direct and/or indirect effects on antibacterial activity. For example, honey produced by *Apis mellifera* honeybees typically has a concentration of sugars of approximately 80 g/100 g (80% w/w). The high concentration of sugars generates a large negative osmotic pressure that can effectively dehydrate a microbial cell [4–6]. The low pH of honey has been shown to denature extracellular and surface proteins that confer membrane stability [7, 8]. In some honeys, hydrogen peroxide (H_2_O_2_) is generated, causing oxidative stress [9]. Lastly, specific molecules such as methylglyoxal (MGO) and bee defensin-1 have also been implicated in the antibacterial activity of some honeys [3, 8]. Each of these factors are likely to play a role in the inhibition or death of bacterial cells.

Honey varies widely in composition, even within similar geographical regions. The primary determinant of honey composition is the bee’s foods source, and in particular the floral nectar [10]. This has led to interest in monofloral honeys and their differing organoleptic, antioxidant and antimicrobial properties [3]. Manuka monofloral honey (from *Leptospermum scoparium*) is particularly well known globally, and may contain concentrations of the antimicrobial component MGO in excess of 800 mg/kg [8, 11].

Honey has a long history as a therapeutic agent and recent studies have demonstrated benefits associated with its use. Clinical trials have already indicated that honey is a useful therapy for some cutaneous bacterial infections, and has a low irritant or allergic potential [2, 12]. It is likely less toxic than antiseptics such as benzalkonium chloride (BAC), chlorhexidine gluconate (CHG) and silver nitrate (AgNO_3_) [13]. Also, since honey has a multifactorial mechanism of action, the development of antibacterial resistance is unlikely and has not been demonstrated to date [1, 3]. Honey also has activity against antibiotic-resistant bacteria, making it an attractive treatment option for infections with antibiotic-resistant organisms such as methicillin-resistant *Staphylococcus aureus* (MRSA) [14]. Additionally, honey may be preferred by some individuals who favour natural products over synthetic agents such as BAC or CHG [15].

Combining honey with other antimicrobial agents, such as antiseptics or essential oils, may have additional benefits compared to the use of honey alone, or antiseptics alone. BAC, CHG, and AgNO_3_ exert antibacterial activity by disrupting the cell membrane [16–18]. In addition, these agents are all cationic, enabling them to interact with the negatively charged bacterial cell wall, leading to destabilisation and cell death [16–18]. Essential oils such as *Melaleuca alternifolia* oil and *Eucalyptus polybractea* oil contain terpenes, which are small, non-polar molecules that can insert into the cell membrane causing loss of cell homeostasis [19, 20]. These antibacterial mechanisms could potentially complement those of honey, resulting in improved overall antibacterial activity. Conversely, there may be decreased antibacterial activity when honey is combined with these topical agents. Antimicrobial interactions between honey and antiseptics or essential oils have not been widely investigated and therefore, in this study we examined the antimicrobial activity of honey when combined with a range of other topically applied antimicrobial agents.

## Methods

### Preparation of honey and antimicrobial reagents

Marri honey (from *Corymbia calophylla*; harvested in Western Australia) and manuka honey with an MGO level of 550 + (harvested in New Zealand) were purchased locally. Honeys were stored at room temperature protected from light. Solutions of honey were prepared at 50% (w/v) in sterile distilled water and were mixed vigorously using a vortex mixer until homogeneous. Each solution was then passed through a glass microfibre prefilter (0.7 µm pore size) to remove large detritus including pollen, then through a 0.2 µm sterilising filter. All honey solutions were used within 1 h of preparation. Tea tree oil (TTO; from *M. alternifolia*) and *Eucalyptus* oil (EPO; from *E. polybractea)* were sourced from Integria Healthcare (Australia), and Bosistos FGB Natural Products (Australia), respectively. Essential oils were stored protected from light at 4 °C. Solutions of TTO and EPO were prepared by diluting each, volume for volume, in a solution of Tween 80 and sterile distilled water in such a way that the final concentration of Tween 80 in all wells was 0.001% v/v. Benzalkonium chloride (BAC; catalogue #12060), chlorhexidine digluconate (CHG; catalogue #C9394) and silver nitrate (AgNO_3_; catalogue #209139) were all sourced from Sigma-Aldrich. Solutions were prepared by dissolving and diluting in sterile distilled water.

### Preparation of inocula

Inocula of *S. aureus* ATCC® 43300 (methicillin-resistant) and *P. aeruginosa* ATCC® 27853 were prepared by suspending morphologically identical colonies from an overnight culture on blood agar in 0.85% saline. The suspension was adjusted to an optical density of a 0.5 McFarland standard (approximately 2 × 10^8^ CFU/mL) using a densitometer. Inocula were further diluted 1 in 50 in 4 × Mueller Hinton Broth (MHB) so that after the addition of 50 µL volumes of inocula to wells of each microtitre plate containing honey and/or antimicrobial agent, the final concentration of broth was 1 × MHB and the concentration of bacteria was approximately 1 × 10^6^ CFU/mL in a final well volume of 200 µL.

### Measurement of fractional inhibitory indices and fixed concentration assays

The antimicrobial relationship between each honey and each agent was investigated using the checkerboard technique, as described elsewhere [21]. In brief, in a 96-well microtitre plate, differing volumes of agent A (marri or manuka honey) were added to wells of each column resulting in descending concentrations, and differing volumes of agent B (BAC, CHG, TTO, or EPO) were added into the wells of each row resulting in descending concentrations. Note that incremental dilutions of 2% (w/v) were used for honeys due to the relatively narrow effective concentration range, whereas serial two-fold dilutions were used for antiseptic agents. If required, volumes of sterile distilled water were added to each well to make the well volumes up to 150 µL. The range of concentrations tested are shown in Supplementary Table 1. Each well was then inoculated with a 50 µL volume of either *S. aureus* ATCC® 43300 or *P. aeruginosa* ATCC® 27853 prepared as previously described to result in final cell concentration of approximately 10^6^ CFU/mL and final well volume of 200 µL. Each microtitre plate was then incubated in ambient air at 37 °C for 24 h.

After incubation, each microplate was visually inspected and inhibitory endpoints, including minimum inhibitory concentrations (MICs) were determined as wells with no visible turbidity. Using the inhibitory endpoints, a fractional inhibitory concentration index (FICI) was determined to summate the relationship between each honey and each antimicrobial agent using a method described previously [22]. FICI values correspond to different interaction types; synergism (FICI ≤ 0.5), additive (0.5 < FICI ≤ 1), indifference (1 < FICI ≤ 2), antagonism (FICI > 2) [22, 23]. The optical density (OD) of each well in all microtitre plates was measured at 600 nm prior to incubation (t = 0; baseline) and again at t = 24 h to quantify relative bacterial growth. Baseline OD values were subtracted from t = 24 h values to generate net OD values. Growth at each concentration of antimicrobial agent was then expressed relative to the positive growth control (100%).

In order to further investigate the relationship between CHG and marri honey at higher concentrations, microbroth dilution assays using fixed concentrations of CHG and 2% increment concentrations (range 2—30% w/v) of marri honey were performed using the previously described broth microdilution assay [23]. The fixed concentrations of CHG were selected based on the MIC values obtained in prior experiments (2 × MIC, and 4 × MIC). All checkerboard and fixed concentration assays were repeated at least twice and on different days.

### Time-kill curves for selected agents

To further examine how other antiseptic agents such as essential oils might potentially interact with the antibacterial activity of honey, selected time-kill studies were conducted. Of the two essential oils, TTO was chosen due to its relatively higher antibacterial activity [24]. Based on preliminary experiments, 0.25% (v/v) TTO and 9% (w/v) marri honey were determined to be optimal concentrations for bacterial killing. Given that *S. aureus* is frequently associated with cutaneous infections it was chosen as the test organism.

To prepare inocula, 1–2 colonies of *S. aureus* ATCC® 43300 were inoculated into 10 ml of trypticase soy broth and incubated at 37 °C with shaking until mid-exponential phase, which was approximately 2.5 h. Cultures were then adjusted to a cell density of approximately 2 × 10^7^ CFU/mL using a densitometer. Varying concentrations of honey and/or TTO were prepared in MHB in 25 mL Erlenmeyer flasks such that after inoculation with 1 ml of adjusted culture, each contained a final concentration of 2 × 10^6^ CFU/mL *S. aureus* ATCC® 43300, antimicrobial agents, and MHB in a total volume of 10 mL. The positive growth control flask contain inoculum in MBH without any antibacterial agent. Each flask was incubated at 37 °C with 120 rpm orbital shaking. At t = 0, 2, 4, and 6 h, cell viability was determined by removing 100µL samples from each flask and serially diluted tenfold in 0.85% saline. A 20 µL volume of each dilution was spot inoculated on Mueller Hinton agar and agar plates were incubated for 24 h at 37 °C, after which colonies were counted to determine the viable cell density [25]. Each time-kill assay was performed three times on different days. Data were log_10_ transformed, then mean and standard deviation values were calculated.

### Statistical analyses

Differences between checkerboard results obtained for marri and manuka honeys were assessed by comparing calculated FICI values for each honey in combination with each agent using one-way ANOVA. Significance was set at *p* < 0.05. Log_10_ transformed viable count data obtained from the time-kill assay was analysed using two-way ANOVA with Tukey’s multiple comparisons test at each time point. Statistical analyses were conducted using GraphPad Prism (Version 8.4.0).

## Results

### Antibacterial activity

Comparison of MICs for marri and manuka honeys demonstrated that both honeys had similar levels of activity against each test organism (Table 1). Comparison of strains showed that *P. aeruginosa* was less susceptible to both honeys compared to *S. aureus*. Similarly, *P. aeruginosa* required higher concentrations of each antimicrobial agent to inhibit growth compared to *S. aureus* for BAC, CHG, AgNO_3_, and EPO. MICs of TTO against *S. aureus* and *P. aeruginosa* were both 1% (v/v).

**Table 1:** MICs of honeys, essential oils and antiseptics

		MIC	
Category	Antibacterial agent	*S. aureus* ATCC® 43300	*P. aeruginosa* ATCC® 27853
Honey	Marri honey	6% w/v	12% w/v
	Manuka honey	6% w/v	14% w/v
Antiseptics	Benzalkonium chloride	8 µg/mL	128 µg/mL
	Chlorhexidine digluconate	2 µg/mL	4 µg/mL
	Silver nitrate	8 µg/mL	16 µg/mL
Essential oils	Tea tree oil	1% v/v	1% v/v
	*E. polybractea* oil	1% v/v	2% v/v

### Antimicrobial interactions

**Fig. 1:**
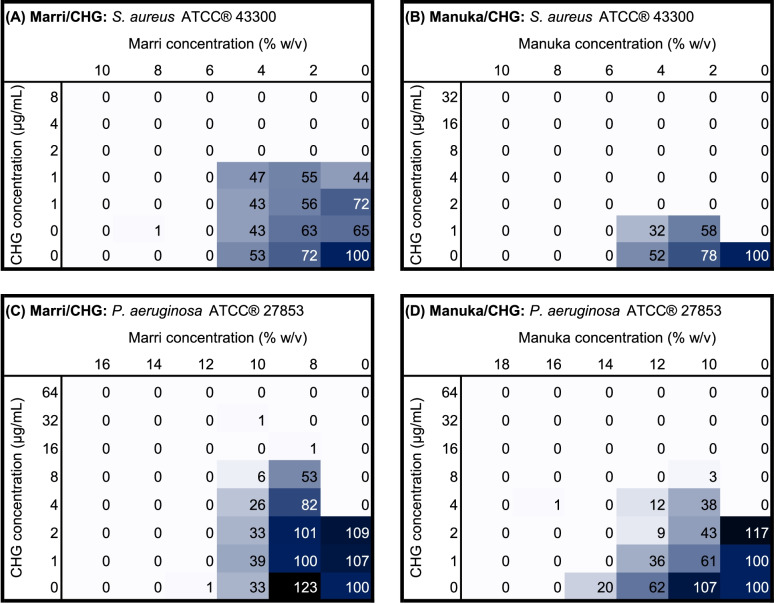
Heat maps indicating mean relative optical density (%) of organisms in checkerboard assays of CHG with either marri honey or manuka honey. Values are expressed as a percentage of the positive growth control. **A** *S. aureus* ATCC® 43300 with CHG/marri honey. **B** *S. aureus* ATCC® 43300 with CHG/manuka honey. **C** *P. aeruginosa* ATCC® 27853 with CHG/marri honey. **D** *P. aeruginosa* ATCC® 27853 and CHG/manuka honey

**Fig. 2:**
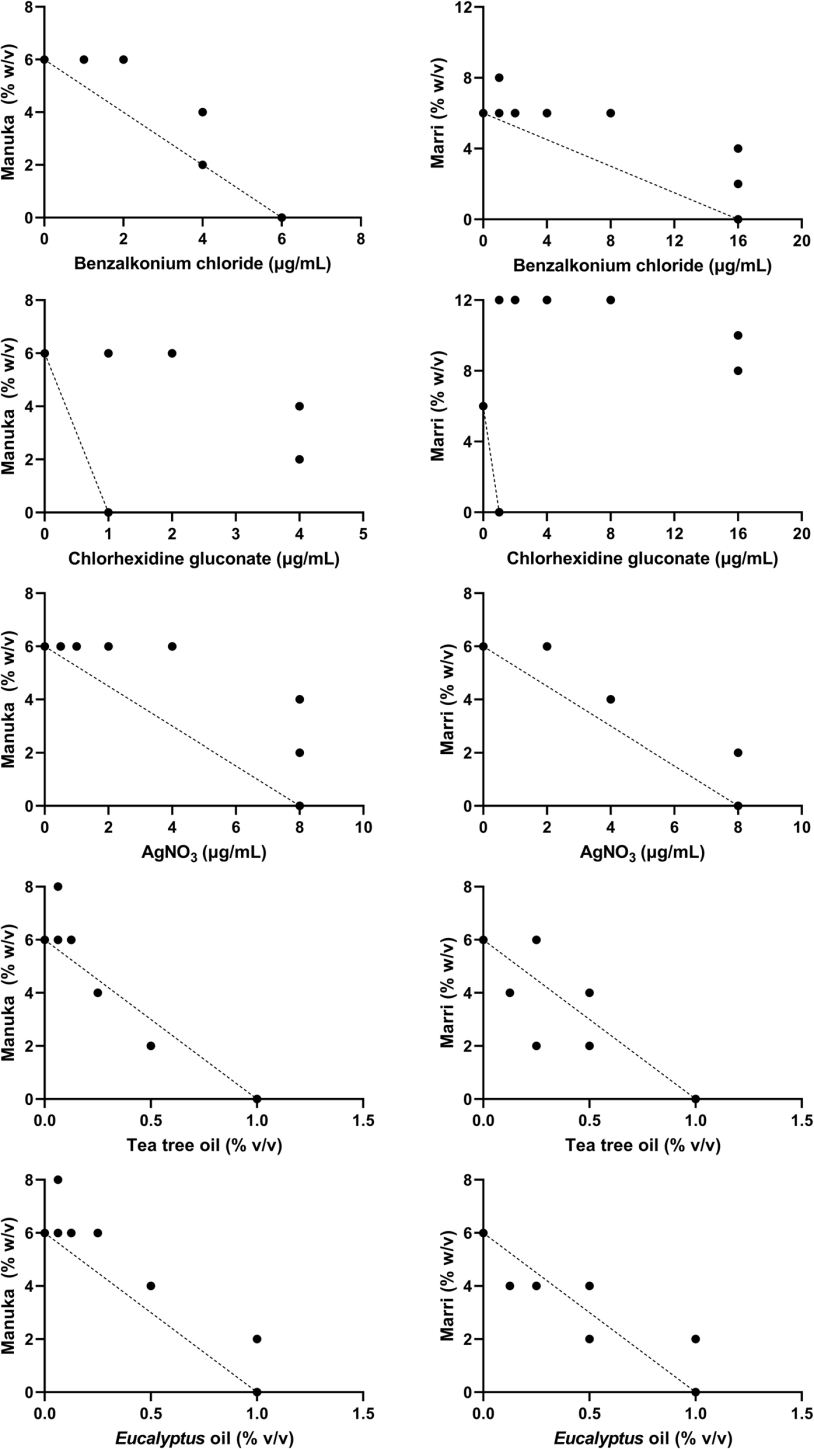
Isobolograms showing the antimicrobial activity of honey (manuka or marri honey) and antiseptic agents (BAC, CHG, AgNO_3_, TTO, and EPO) against *S. aureus* ATCC® 43300. Dotted line represents an additive effect

**Fig. 3:**
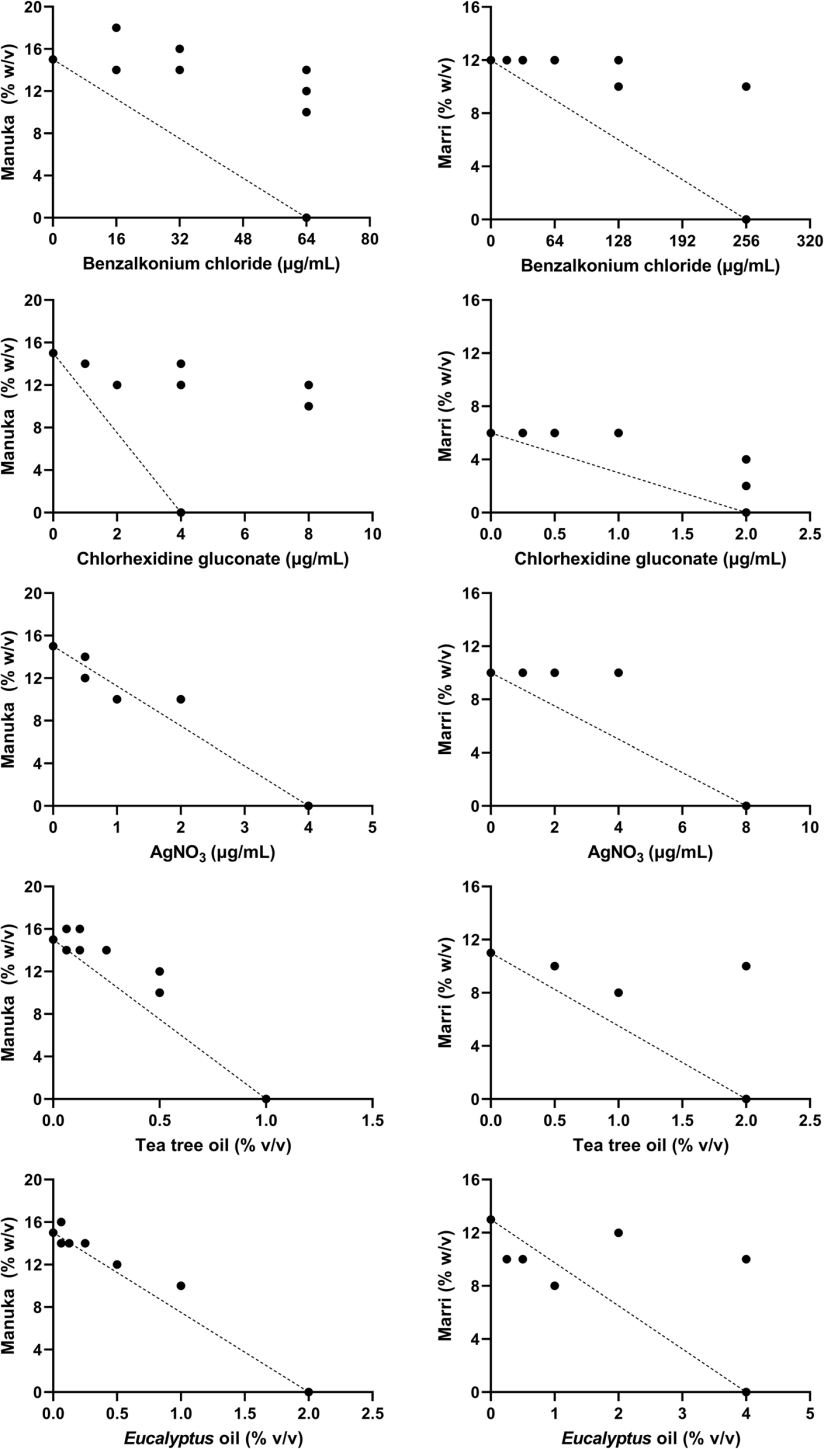
Isobolograms showing the antimicrobial activity of honey (manuka or marri honey) and antiseptic agents (BAC, CHG, AgNO_3_, TTO, and EPO) against *P. aeruginosa* ATCC® 27853. Dotted line represents an additive effect

Antimicrobial synergy testing using checkerboard assays indicated mostly additive or indifferent relationships betwen agents, with FICI values ranging mostly between 0.5 and 2 (Table 2). Marri and manuka honeys showed significant differences in their interactions with antiseptic agents, however no consistent trends were identified. Figure 1 shows heatmaps of CHG and honey, the combination of which showed some antagonistic effect. Modest synergy was observed between TTO and marri honey against *S. aureus* at a concentration of 0.5% v/v and 2% w/v respectively (FICI 0.46). The full extent of the antimicrobial interactions are best shown on isobolograms (Fig. 2 and Fig. 3). The isobolograms show that most relationships approximate additivity, which is reflected in Table 2. Fixed-concentration assays of CHG and marri honey showed an antagonistic effect for *P. aeruginosa*, in agreement with the antagonism observed in the checkerboard assay (Table 3).

**Table 2:** Calculated FICI values for each honey/antiseptic combination. *P* values indicate significant difference between marri and manuka honey for each antiseptic against each organism

		Median FICI (range)				
	Agent	Marri Honey		Manuka Honey		*p* value†
*S. aureus* ATCC® 43,300	BAC	1.25	(0.88—1.67)	1.42	(1.25—1.67)	0.0542
	CHG	2.00	(1.25—4.83)	2.33	(2.00—2.67)	0.5438
	AgNO_3_	0.92	(0.83—1.13)	1.29	(1.06—1.67)	0.0019**
	TTO	0.90	(0.46—1.13)	1.15	(0.83—1.46)	0.0325*
	EPO	0.90	(0.58—1.25)	1.21	(1.06—1.40)	0.2024
*P. aeruginosa* ATCC® 27853	BAC	1.25	(1.06—1.83)	1.71	(1.25—2.00)	0.0030**
	CHG	1.33	(1.13—1.67)	1.88	(1.25—2.75)	0.0058**
	AgNO_3_	1.25	(1.13—1.50)	0.98	(0.88—1.21)	0.0051**
	TTO	1.42	(1.33—1.67)	1.13	(1.06—1.36)	0.0011**
	EPO	1.17	(0.96—1.38)	1.05	(0.94—1.21)	0.1451

^†^ indicates significant differences between marri and manuka FICI values. *p<0.05, **p<0.01

*BAC* Benzalkonium chloride, *CHG* Chlorhexidine digluconate, *AgNO*_*3*_ Silver (I) nitrate, *TTO* Tea tree oil, *EPO* *Eucalyptus polybractea* oil

**Table 3:** MIC values of marri honey in broth microdilution assays in the presence of fixed concentrations of chlorhexidine digluconate (CHG)

Organism	Agent/combination	MIC	
		Honey (% w/v)	CHG (µg/mL)
*S. aureus* ATCC® 43300	CHG		2
	Marri honey	6	
	Marri honey + CHG 4 µg/mL	4	
	Marri honey + CHG 8 µg/mL	< 2	
*P. aeruginosa* ATCC® 27853	CHG		8
	Marri honey	12	
	Marri honey + CHG 16 µg/mL	16	
	Marri honey + CHG 32 µg/mL	16	

### Time-kill assay

**Fig. 4:**
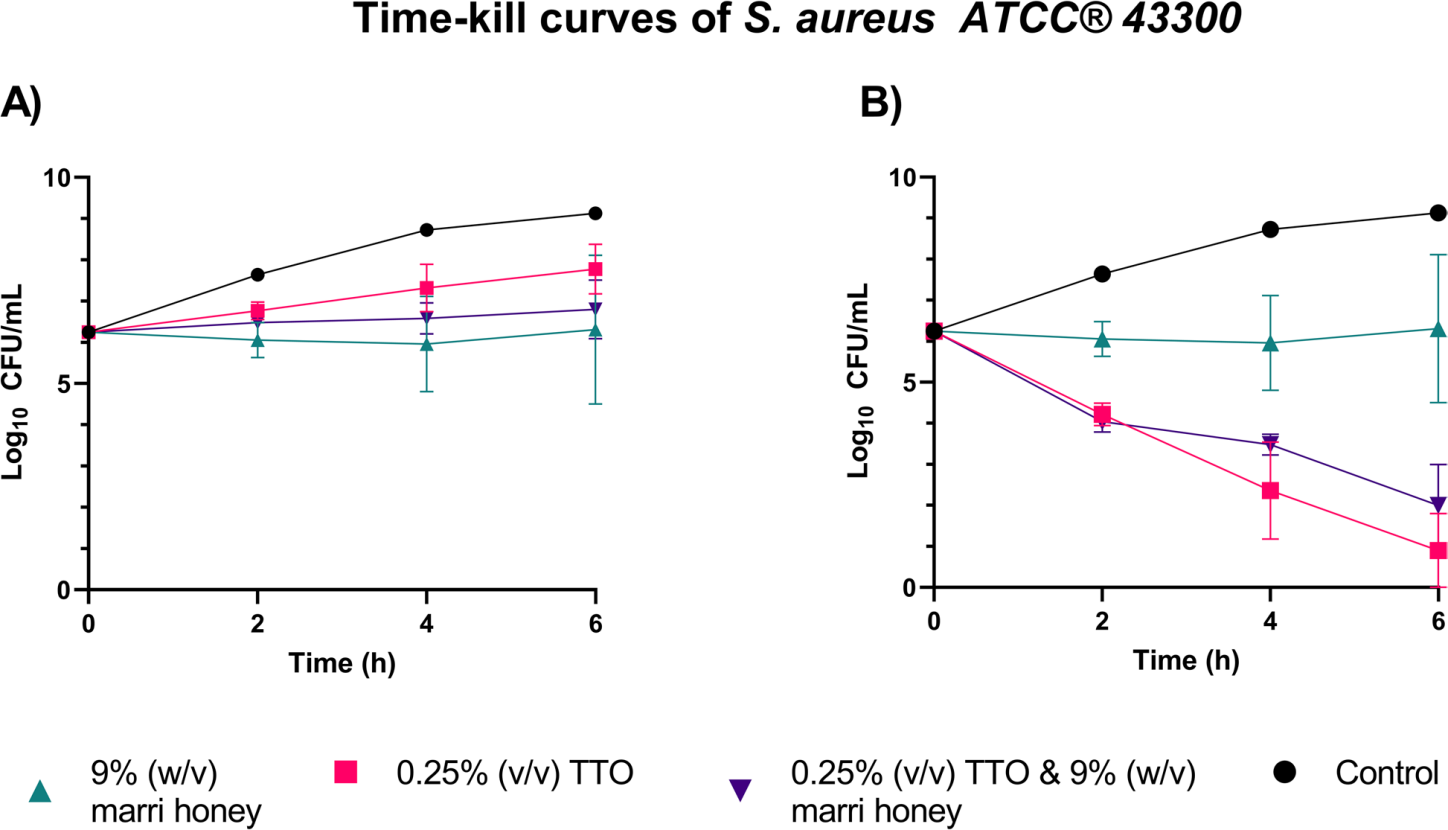
Time-kill curves of *S. aureus* ATCC® 43300 in the presence of either marri honey, TTO, or both. Error bars show standard error

Further investigation of antimicrobial relationships using time-kill assays demonstrated > 3 Log^10^ CFU/mL reductions in *S. aureus* ATCC® 43300 after 6 h in the presence of both 0.25% (v/v) TTO and 0.25% (v/v) TTO/9% (w/v) marri honey combination, but not 9% (w/v) marri honey alone (Fig. 4). There was a significant difference in the viable cell population at t = 6 h between all treatments and the control (*p* < 0.05), as well as between marri honey alone and tea tree oil alone or in combination with marri honey (*p* < 0.0001 and *p* < 0.001, respectively).

## Discussion

This study investigated the in vitro antibacterial interactions between two monofloral honeys and several topical antiseptic agents. The majority of antibacterial interactions observed were classified as indifferent or additive, with FICI values of between 0.5 and 2. This indicates that in vitro, for the most part, there are no antagonistic or synergistic interactions between either of the honeys and the various antiseptic agents tested. Marri honey was also shown to have an indifferent interaction with TTO in the time kill assay, with no significant difference in the viability of cultures exposed to TTO alone compared to TTO combined with marri honey.

Whilst most antibacterial interactions were indifferent, in some instances FICI values > 2 were observed, indicating potential antagonism. The extent of the antagonistic relationship is shown clearly in Figs. 2 and 3 alongside the mostly additive relationships seen with other antiseptics. As seen in the figures, this antagonistic relationship was most profound when honey was combined with CHG. The antibacterial mechanisms of action of both honey and CHG are complex and multifaceted, meaning that there may be multiple potential mechanisms by which antagonism may be occurring [3, 26]. Furthermore, the antagonistic interaction between honey and CHG may occur abiotically in the aqueous culture medium (independent of the bacterial cells), on the bacterial cell surface, inside the bacterial cell, or may be a combination of any of these. CHG is known to be incompatible with a number of compounds, and its bioavailability may be reduced by the formation of insoluble salts or by incorporation into micelles [27]. It is therefore possible that one or more honey components may form precipitates with CHG. In addition, the pH of honey, which is typically between 4—5, may affect the stability and/or efficacy of CHG, as the optimal pH range for CHG antibacterial activity is stated as 5.5—7 [27]. That said, the change in MIC of CHG when combined with honey was relatively minor and given that the typical concentrations of chlorhexidine in therapeutic products are many orders of magnitude higher (10–40 mg/mL) than the MIC values (4 µg/mL), clinical failure due to antibacterial antagonism would be unlikley to occur [28]. Additional studies are required to elucidate the specific mechanism of antagonism between honey and CHG, and to determine the therapeutic relevance (if any) of the interaction.

Comparison of marri and manuka honeys in terms of how each interacted with the antiseptics agents did not reveal any global trends. When checkerboard data for each type of honey were compared, there were several isolated instances where honeys differed significantly in terms of interactions with an antiseptic or a specific organism, but there were no obvious, overarching trends observable between organisms, honeys, or agents. This suggests that the two different honey types do not interact with antiseptics in a fundamentally different manner. This is interesting because manuka and marri honeys vary in both their active antibacterial compounds and corresponding mechanisms of antibacterial action. In manuka honey, MGO is said to contribute substantially to activity but hydrogen peroxide does not, whereas in marri honey the opposite occurs as MGO is absent and hydrogen peroxide activity is typically present [29, 30]. Despite these differences, the two honeys showed similar levels of activity against each organism when tested alone. Comparison of test organisms showed that *P. aeruginosa* was consistently less susceptible to both honeys compared to *S. aureus*, a finding that is consistent with previous data [29]. MICs obtained for the other antiseptic agents were also largely consistent with previously published data [27, 31, 32].

Antimicrobial interactions between specific honeys and a wide range of other natural products, including some essential oils, have been investigated previously [33]. Some have shown synergistic or additive interactions, however, in many instances these synergistic relationships were reported based on results from agar diffusion assays and were not confirmed by checkerboard or time kill assays. A study investigating interactions between 12 honeys and *Origanum vulgare* essential oil against four different bacterial species using a checkerboard assay found largely indifferent or additive interactions with only a few isolated instances of synergy [34], which is in agreement with findings from the current study. Combinations of honey and antiseptics do not appear to have been investigated previously, whereas interactions between honey and antibiotics have, with rifampicin and oxacillin two antibiotics shown to have synergistic activity with honey [35–37].

Results from this study suggest that concomitant therapy with honey and products containing AgNO_3_, BAC, TTO, or EPO is unlikely to impact the antimicrobial activity of any of the agents. Although synergistic interactions were not found, combination therapy utilising honey and an antiseptic may have a role in the treatment of some superficial infections due to the inherent advantages of combination therapies, including the potential to stem the development of antimicrobial resistance [38, 39]. For example, honey could be combined with a silver-impregnated dressing to allow for controlled release of honey into the wound. This combination has not been reported for honey, however augmentation of silver-impregnated dressings with chlorhexidine has been demonstrated in vitro [40]. The potential remains for honey to be combined with antiseptics for topical therapeutic use.

## Conclusions

In summary, this study has demonstrated that for most combinations of antiseptics and honey there is no impact on antimicrobial activity. The exception is CHG, which may have an antagonistic antimicrobial relationship with honey. This indicates that honey-based treatments may be compatible with other topical agents such as AgNO_3_. Future research is required to validate this data and to expand the scope by testing additional types of honeys, antiseptics and bacterial organisms, including antibiotic resistant clinical strains.

## Supplementary Information

**Additional file 1: Supplementary Table 1.** Ranges of agents utilised in the checkerboard assays to determine interactions between honeys and other antimicrobials.

## Data Availability

The datasets used and/or analysed during the current study are available from the corresponding author on reasonable request.

## Abbreviations

AgNO_3_: Silver (I) nitrate
ATCC®: American Type Culture Collection
BAC: Benzalkonium chloride
CHG: Chlorhexidine digluconate
EPO: *Eucalyptus **polybractea* Oil
FICI: Fractional inhibitory concentration index
H_2_O_2_: Hydrogen peroxide
MGO: Methylglyoxal
MIC: Minimum inhibitory concentration
MRSA: Methicillin resistant *Staphylococcus aureus*
OD: Optical density
TTO: *Melaleuca alternifolia* (Tea tree) oil
