# Supplementary material for: Antibacterial interactions between two monofloral honeys and several topical antiseptics, including essential oils
Source: BMC Complement Med Ther. 2022 Aug 26;22:228. doi: 10.1186/s12906-022-03695-x (PMC9419324; doi:10.1186/s12906-022-03695-x)
Supplement: Supplementary file 1 — Additional file 1: Supplementary Table 1. Ranges of agents utilised in the checkerboard assays to determine interactions between honeys and other antimicrobials. [file 12906_2022_3695_MOESM1_ESM.docx]

**Supplementary Table 1** Ranges of agents utilised in the checkerboard assays to determine interactions between honeys and other antimicrobials.

|  | ***S. aureus* ATCC® 43300** | | ***P. aeruginosa* ATCC® 27853** | |
| --- | --- | --- | --- | --- |
| **Agent** | **Min.** | **Max.** | **Min.** | **Max.** |
| Marri honey (% w/v) | 2 | 10 | 10 | 18 |
| Manuka honey (% w/v) | 2 | 10 | 10 | 18 |
| Benzalkonium chloride (µg/mL) | 1 | 32 | 16 | 512 |
| Chlorhexidine gluconate (µg/mL) | 1 | 32 | 4 | 128 |
| Silver nitrate (µg/mL) | 0.5 | 32 | 0.5 | 32 |
| Tea tree oil (% v/v) | 0.25 | 4 | 2 | 10 |
| Eucalyptus oil (% v/v) | 0.25 | 4 | 2 | 10 |
